# Stochastic and deterministic assembly processes of microbial communities in relation to natural attenuation of black stains in Lascaux Cave

**DOI:** 10.1128/msystems.01233-23

**Published:** 2024-01-30

**Authors:** Zélia Bontemps, Yvan Moënne-Loccoz, Mylène Hugoni

**Affiliations:** 1 UMR 5557 Ecologie Microbienne, Université Claude Bernard Lyon 1, CNRS, INRAE, VetAgro Sup, Villeurbanne, France; 2 Department of Medical Biochemistry and Microbiology, Science for Life Laboratories, Uppsala University, Uppsala, Sweden; 3 UMR 5240 Microbiologie Adaptation et Pathogénie, INSA Lyon, CNRS, Université Claude Bernard Lyon 1, Villeurbanne, France; 4 Institut Universitaire de France (IUF), France; Ocean University of China, Qingdao, China

**Keywords:** community assembly processes, disturbance, rare biosphere, microbial ecology

## Abstract

Community assembly processes are complex and understanding them represents a challenge in microbial ecology. Here, we used Lascaux Cave as a stable, confined environment to quantify the importance of stochastic vs deterministic processes during microbial community dynamics across the three domains of life in relation to an anthropogenic disturbance that had resulted in the side-by-side occurrence of a resistant community (unstained limestone), an impacted community (present in black stains), and a resilient community (attenuated stains). Metabarcoding data showed that the microbial communities of attenuated stains, black stains, and unstained surfaces differed, with attenuated stains being in an intermediate position. We found four scenarios to explain community response to disturbance in stable conditions for the three domains of life. Specifically, we proposed the existence of a fourth, not-documented yet scenario that concerns the always-rare microbial taxa, where stochastic processes predominate even after disturbance but are replaced by deterministic processes during post-disturbance recovery. This suggests a major role of always-rare taxa in resilience, perhaps because they might provide key functions required for ecosystem recovery.

**IMPORTANCE:**

The importance of stochastic vs deterministic processes in cave microbial ecology has been a neglected topic so far, and this work provided an opportunity to do so in a context related to the dynamics of black-stain alterations in Lascaux, a UNESCO Paleolithic cave. Of particular significance was the discovery of a novel scenario for always-rare microbial taxa in relation to disturbance, in which stochastic processes are replaced later by deterministic processes during post-disturbance recovery, i.e., during attenuation of black stains.

## INTRODUCTION

The relevance of community assembly rules for predicting and modeling how communities face different environmental scenarios, based on events controlling the presence and abundance of species in a community, is a key issue in ecology. According to the niche-based theory, deterministic processes linked to abiotic and biotic factors dictate community assembly, making environmental filtering and/or niche selection the prevalent mechanisms underpinning ecological selection (1, 2). Conversely, the neutral theory emphasizes the role of stochastic processes (1–5), with a major part played by ecological drift, dispersal limitation, and probabilistic dispersal (2, 6). While the consensus is that both deterministic and stochastic processes simultaneously influence the assembly of communities, along a continuum (2, 5), the challenge is now to quantify their relative importance in various ecosystems. While these ecological concepts have been historically applied to macroorganisms (7–12), they have been much less considered for microbial communities (13–16).

In natural ecosystems, microorganisms are highly diversified, growing and evolving fast, depending on horizontal gene transfers or mutation (17), and they form complex assemblages of Archaea, Bacteria, and Microeukaryota. Most studies have focused on a single domain of life instead of taking into account the entire microbiome (16, 18–21). As key contributors to biogeochemical cycles, microorganisms represent a biosphere compartment that must be considered to model and predict global changes (22). Most often, this has been done with dominant microorganisms or entire communities, but in recent years, the functional importance of less prevalent taxa has been increasingly recognized (23). This led to the formal definition of two types of rare taxa, namely the always-rare taxa (i.e., with very small relative abundance in all samples) and the conditionally rare taxa (i.e., with a relative abundance that is low in some samples and very low in others) (24–26). Indeed, members of the rare biosphere can be metabolically active and contribute significantly to ecosystem functioning (23, 27). Some of these rare taxa may become occasionally abundant under particular environmental conditions (28), accounting for large temporal shifts in microbial structure (29, 30).

Microbial communities exposed to disturbance may display (i) resistance, involving plasticity (31); (ii) resilience, based on functional redundancy (32, 33), and/or (iii) an alternative state where new taxa harboring different functions arise, inducing a new community functioning (33, 34). Many studies focusing on microbial responses to disturbance have used experimental setups that lacked realism and complexity (i.e., lab conditions and simplified consortia), but stable ecosystems experiencing ecological disturbance are more relevant to assessing community assembly patterns in natural conditions.

Cave ecosystems provide a unique set of stable environmental variables, including the absence of light, high relative humidity and CO_2_ concentrations, almost constant temperature, and oligotrophy (0.5 mg organic carbon/L) (35–44). Interestingly, many are rather confined, implying slow exchanges with surrounding karst (45). This is the case today for Lascaux Cave (Montignac-Lascaux, Dordogne, France), which was discovered in 1940 and opened to the public in 1948. Infrastructure work, high visitor numbers, and excessive chemical treatments led to cave imbalance and artwork conservation issues, and the cave was closed in 1963 (46). From this time, the cave has been monitored from a climatic, hydrogeological, and microbiological standpoint (45–49). Within caves, terminal rooms are particularly stable, such as Lascaux’s Chamber of Felines. During the 2000s, Lascaux Cave was the theater of chemical treatments [especially benzalkonium chloride; (50)] to prevent fungal outgrowth problems related to past tourism activities (46, 51). This anthropization constituted a disturbance for microbial communities initially established, prompted the development of melanin-producing fungi on cave surfaces, and resulted in the formation of black stains, many still present (46, 48–50, 52). Lascaux’s Chamber of Felines was never visited by the public, yet it underwent black stain formation and was treated with chemical biocides (but less than elsewhere).

**Fig 1:**
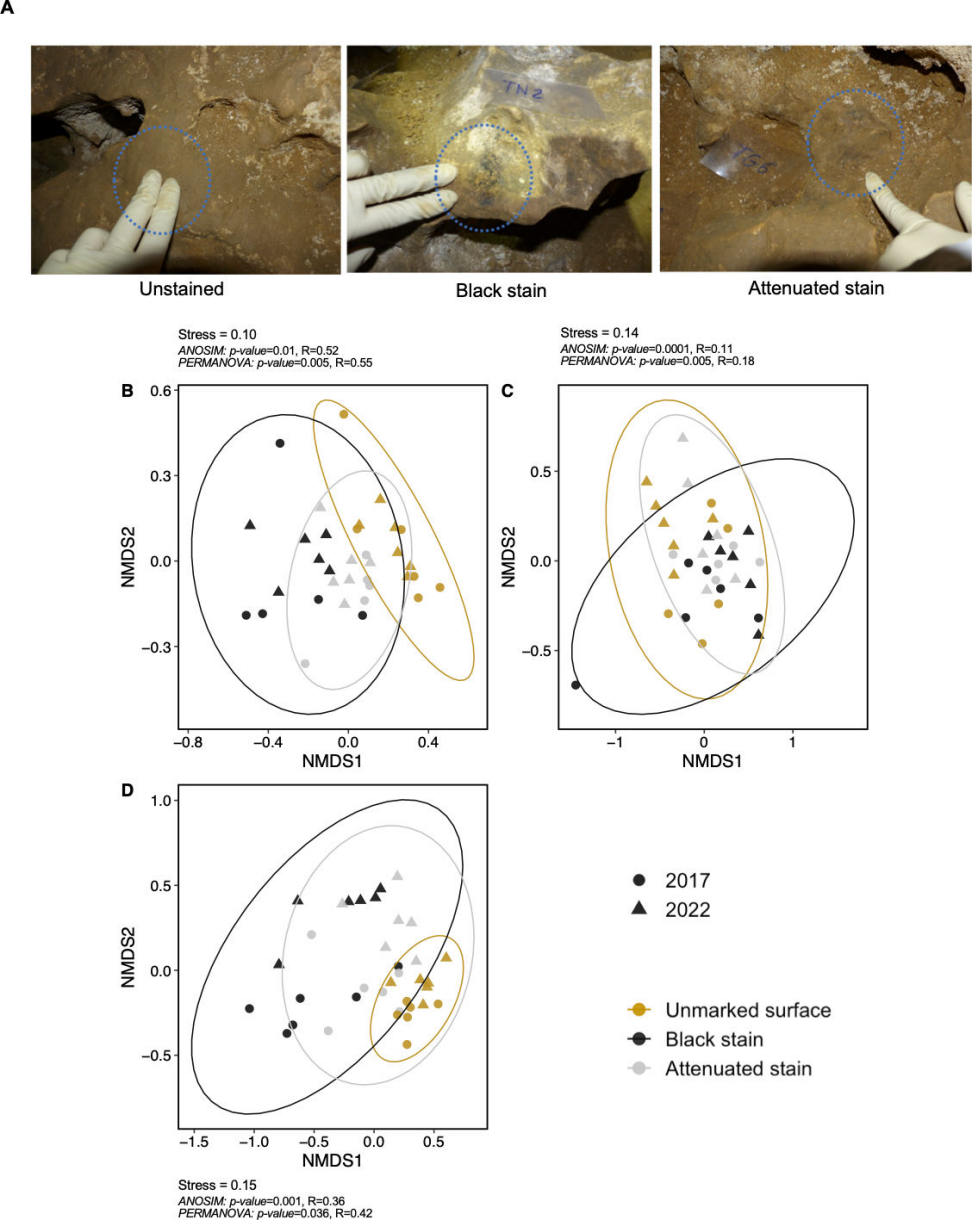
Comparison of rock surface conditions in Lascaux’s Chamber of Felines. (**A**) Photographs of unstained limestone (resistant microbial community), black stain (impacted community), and attenuated stain (resilient status) (credit: DRAC Nouvelle Aquitaine). (**B–D**) Differences in bacterial (**B**), archaeal (**C**), and microeukaryotic communities (**D**) indicated by non-metric dimensional scaling (NMDS) ordination based on Bray-Curtis dissimilarity index calculated on abundance matrices at the operational taxonomic unit level. Permutational analysis of variance (PERMANOVA), stress value, and analysis of similarities (ANOSIM) are presented in each case.

In the Chamber of Felines, three rock surface conditions (Fig. 1) are present on the same walls, i.e., (i) limestone that has never been stained (microbial community considered resistant to anthropization; control), (ii) black stains present since 2002 (impacted community), and (iii) former black stains, now grayish, where black pigmentation has progressively disappeared since 2007. These grayish, attenuated stains differ little from unstained limestone and correspond to a resilient status, probably involving particular microbial dynamics for all three domains of life. Here, we aimed to test the hypothesis of similar community assembly patterns for Archaea, Bacteria, and Microeukaryota retrieved during the resistant, impacted, and resilient states. By assessing the abundant and rare biospheres, we demonstrated that always-rare taxa, shaped by stochastic processes in control and impacted communities, were the only microbial fraction selected among the three domains of life during the resilient state.

## RESULTS

### Comparison of black stains, attenuated stains and unstained surfaces

**Fig 2:**
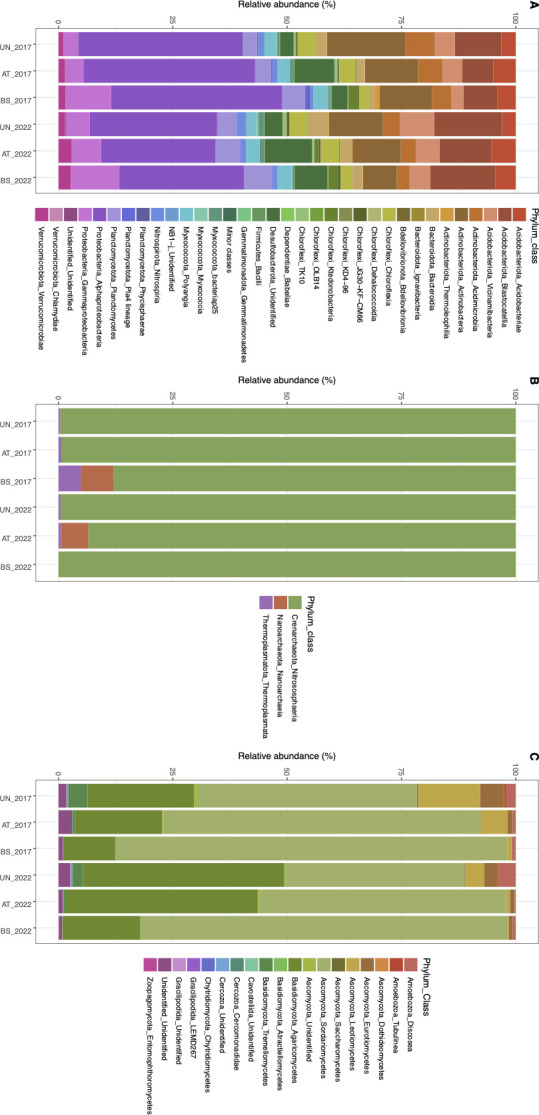
Comparison at phylum level of microbial communities on unstained limestone (resistant microbial community), black stain (impacted community), and attenuated stain (resilient status) in Lascaux’s Chamber of Felines. The analysis was done with bacteria (**A**), archaea (**B**), and microeukaryotes (**C**). Bacterial classes representing less than 0.1% are combined in the minor phyla category.

Non-metric multidimensional scaling (NMDS) (Fig. 1) and permutational multivariate analysis of variance (Table S1) showed that microbial communities in the three rock surface conditions differed, but the differences between sampling dates were not significant. Based on community composition data for phyla, attenuated stains displayed an intermediate situation between black stains and unstained surfaces, but with variability between 2017 and 2022 (Fig. 2). For example, Gammaproteobacteria, Vicinamibacteria, and Actinobacteria represented, respectively, 4.2%, 6.0%, and 14.5% of the bacterial sequences on unstained surfaces and 10.2%, 3.5%, and 9.4% in black stains, with intermediate values of, respectively, 5.2%, 4.8%, and 11.1% in attenuated stains (Fig. 2). The same trend was observed for the microeukaryotic community, especially for the Sordariomycetes, Agaromycetes, and Letiomycetes with, respectively, 43.5%, 33.6%, and 8.9% of sequences on unstained surfaces and 82.9%, 14.2%, and 0.5% in black stains vs 61.7%, 30.5%, and 3.3% in attenuated stains, respectively (Fig. 2). It was also the case for composition data at genus level for bacteria and microeukaryotes, and variability was again evidenced when comparing sampling dates (Fig. S1).

For each taxonomic marker, differences in diversity indices (Chao-1, Shannon, and Simpson) between sampling dates were not significant (multiple comparisons with Tukey tests: all *P* > 0.1) (Table S2). However, each of the three rock surface conditions was significantly distinct based on the Chao-1 index for the bacterial data set (*P* = 0.013) and all diversity indices for the microeukaryotic data set (all *P* < 0.05). No differences were observed for the archaeal data set (all *P* > 0.1).

### Quantification of the relative importance of assembly processes at whole community level

**Fig 3:**
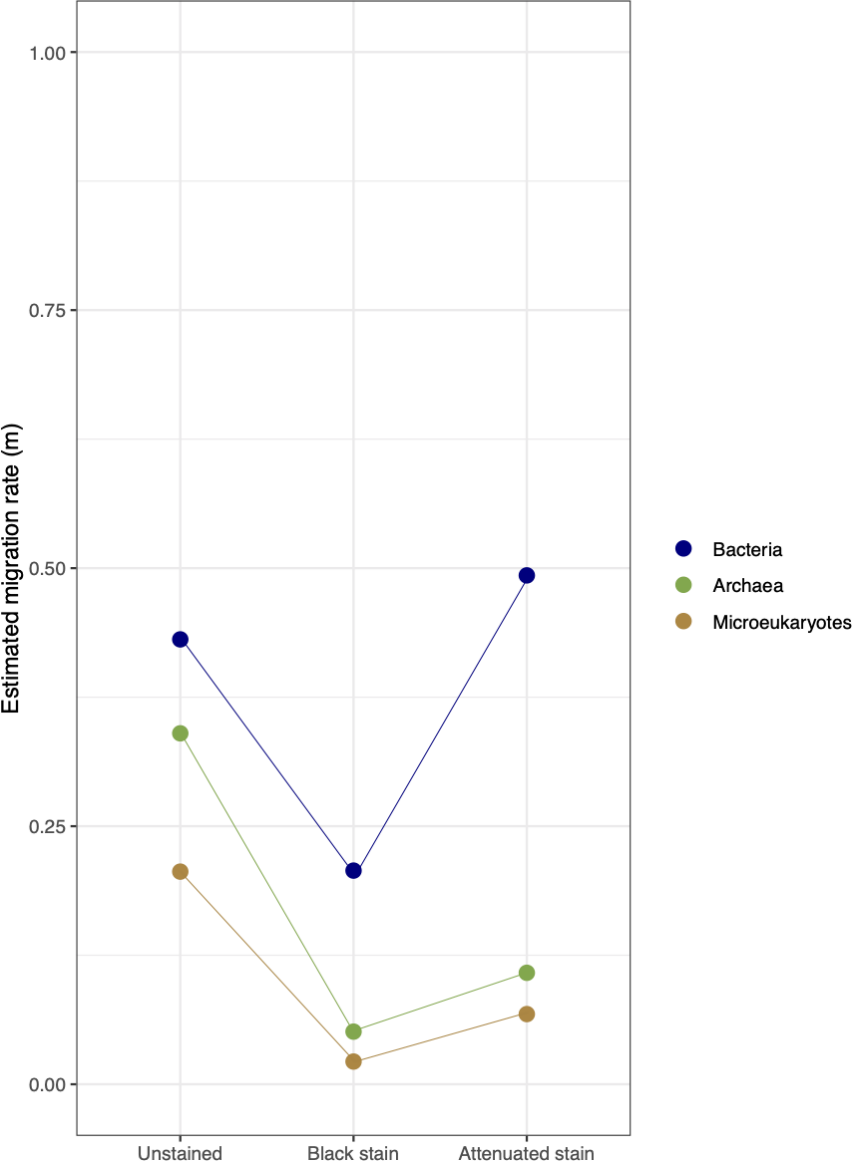
Estimated migration rate (*m*) of the neutral model during microbial dynamics for the three domains of life. The goodness-of-fit of the neutral model and the comparison of the maximum likelihood fit of the neutral, binomial, and Poisson models for each community can be found in Fig. S1 and Table S1.

Community assembly patterns were evaluated to understand the relative importance of stochastic vs deterministic processes using neutral-community model (NCM). The NCM fitted well for archaeal, bacterial, and microeukaryotic communities (Fig. S2), outperforming binomial and Poisson distribution models (Table S3). This points to the prevalence of stochastic processes (passive dispersal or ecological drift) independently of the random sampling performed within each of the three rock surface conditions. For the three domains of life combined, the community migration rate (*m*) reached an average of 0.47 in resistant communities vs 0.06 in impacted communities and 0.19 in resilient communities (Fig. 3), pointing to more extensive dispersal and ecological drift in resistant communities compared with the others.

**Fig 4:**
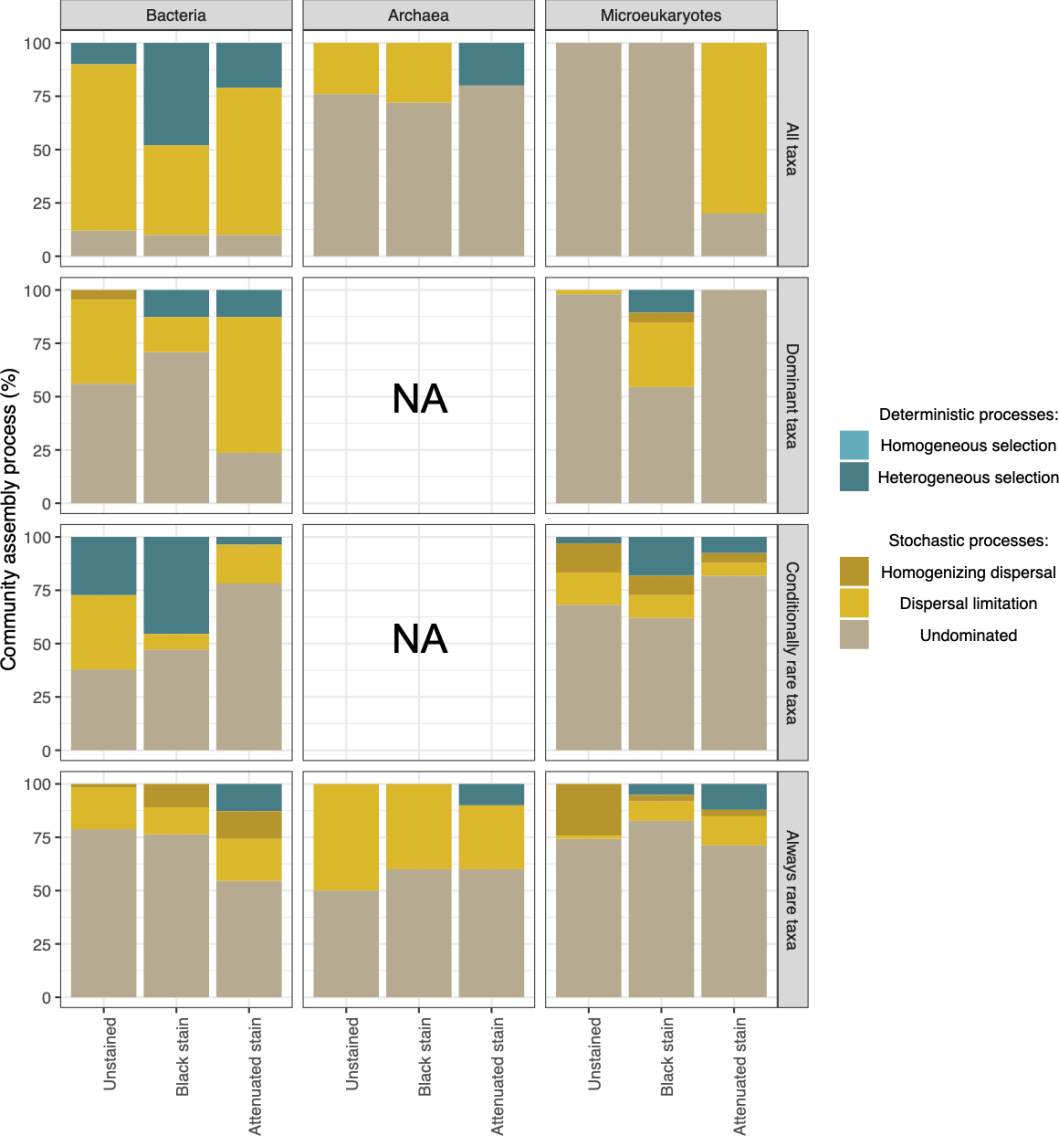
Contribution of deterministic and stochastic processes for all taxa, dominant taxa, conditionally rare taxa, and always-rare taxa of the three microbial communities. Moderate taxa were not assessed because the number of OTUs was too small. Community assembly processes were calculated for deterministic processes (including homogeneous and heterogeneous selection) and stochastic processes (including homogenizing dispersal, dispersal limitation, and undominated processes). NA, not available.

We quantified the relative contribution of stochastic and deterministic processes shaping microbial community assembly by calculating the β-nearest taxon index (βNTI) (Fig. S3) and Bray-Curtis-based Raup-Crick (RC_bray_) (Fig. S4) from the abundance of operational taxonomic units (OTUs) and phylogenetic distances. The βNTI is the difference between the observed β-mean nearest taxon index (βMNTD) and the mean of the null distribution of βMNTD normalized by its standard deviation. βNTI values > +2 indicate significantly more phylogenetic turnover than expected (heterogeneous selection), and βNTI values <−2 indicate less phylogenetic turnover than expected (homogeneous selection) (53). As the observed βMNTD value did not significantly deviate from the null βMNTD distribution (i.e.*,* |βNTI| < 2), it meant stochastic turnover in phylogenetic composition. Since this stochasticity could entail dispersal limitation, homogenizing dispersal, or the lack of a single dominant process (termed the undominated fraction) (13), this was clarified by calculating the RC_bray_ on pairwise comparisons with |βNTI| < 2 as follows: |RC_bray_| > +0.95 means dispersal limitation, |RC_bray_| < −0.95 means homogenizing dispersal, and −0.95 < |RC_bray_| < 0.95 means the undominated fraction (2, 13, 54). The identified assembly processes showed contrasted patterns among the three domains of life. Indeed, the contribution of stochastic processes to bacterial community assembly amounted to 93.3% on unstained limestone and 80.0% in attenuated stains vs only 53.3% in black stains (Fig. 4). More precisely, the entire bacterial community showed a high level of heterogeneous selection in black stains (48.8%) but exhibited dispersal limitation in attenuated stains (69.2%) and on unstained surfaces (78.3%). For the archaeal community, in contrast, stochastic processes accounted for 100.0% of community assembly on unstained limestone and black stains with 24.5% and 28.0% of dispersal limitation, respectively. Whereas heterogenous selection processes accounted for 20.8% in attenuated stains. Assembly patterns were exclusively stochastic for the microeukaryotic community, regardless of rock surface condition with 100% of undominated processes (i.e., selection was too weak and dispersal rates not strong enough for either process to drive compositional turnover) in both unstained and black stain conditions and 80.4% of dispersal limitation in attenuated stain rock surface condition.

### Assembly processes for abundant vs rare biosphere

To go beyond analyses considering the microbial community as a whole, the abundance of taxa (i.e.*,* abundant vs rare biosphere) was also taken into account for each domain of life to decipher community assembly patterns in more detail. To this end, OTUs were classified using four established abundance categories advocated for that purpose (55, 56), i.e., the “always-rare” taxa, the “conditionally rare” taxa, the “moderate” taxa, and the “dominant” taxa (Fig. S5), and assembly processes were reassessed for each taxa category. Bacterial dominant taxa showed a high level of heterogeneous selection in black stains (66.7%) but exhibited dispersal limitation in attenuated stains and on unstained surfaces. In contrast, microeukaryotic dominant taxa mostly displayed stochastic properties, with mainly undominated processes on the unstained surfaces and in black stains but only dispersal limitation in the attenuated stains. However, heterogeneous selection also occurred in impacted communities (33.3%), as for Bacteria. Analysis was not possible with Archaea, as only two dominant taxa were found.

The moderate taxa could not be analyzed because the number of OTUs for this category was less than six in each of the three microbial communities (see Materials and Methods).

In conditionally rare taxa, dispersal limitation predominated for Bacteria (75.5%) and microeukaryotes (60.0%) in all three surface conditions, whereas such taxa were rare in Archaea. Heterogeneous selection also contributed to the assembly of Bacteria (in all conditions) and microeukaryotes (but only in black stains).

On unstained surfaces and in black stains, always-rare taxa from the three domains of life were associated to stochastic assembly, mostly by undominated processes. The latter means that the underlying assembly mechanisms are not quantifiable yet, probably because the rare biosphere of caves remains very poorly documented. The same trend was found in the attenuated stains, with also a contribution of deterministic processes amounting to 6.7%, 10.0%, and 26.7% for heterogeneous selection in microeukaryotic, archaeal and bacterial communities, respectively (Fig. 4). This result clearly shows that always-rare taxa from the three domains of life were selected only in the resilient status, suggesting their importance in ecosystem functioning.

### Always-rare taxa composition across rock surface conditions

**Fig 5:**
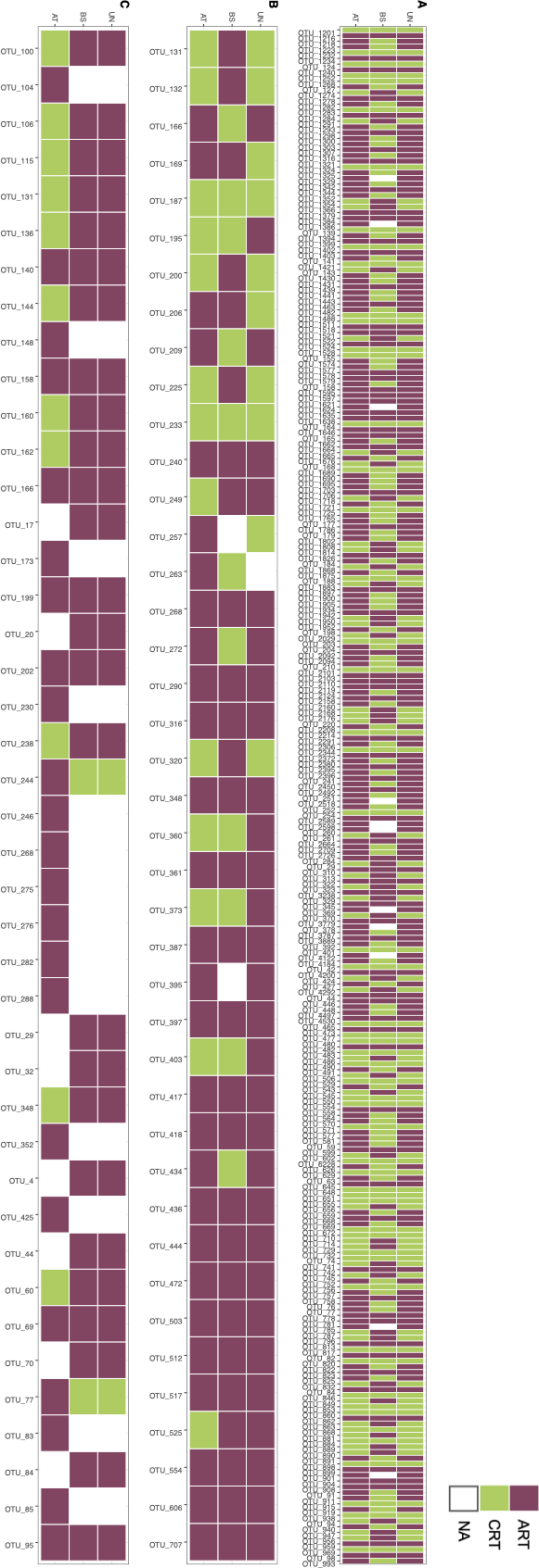
Distribution of always-rare taxa affiliated with Bacteria (A), Archaea (B), or microeukaryotes (C) for each rock surface condition. The heatmap was performed using OTU abundance of always-rare taxa (represented with a yellow-red gradient) and conditionally rare taxa (in blue).

We investigated the taxonomic identity of the 286 always-rare taxa since they were the only OTU category that had undergone deterministic assembly processes in attenuated stains. Among them, 150 OTUs displayed the always-rare status in all three rock surface conditions and 136 in one or two rock surface conditions (Fig. 5). Of these 286 OTUs, 15 were retrieved only in attenuated stains and they belonged to Acidobacteria (three OTUs), Actinobacteria (three OTUs), Chloroflexi, Alphaproteobacteria, Planctomycetota, Thaumarchaeota, and Ascomycota. In addition, 42 other OTUs, including Actinobacteria (12 OTUs), Alphaproteobacteria (5 OTUs), and Ascomycota (20 OTUs), were common to attenuated stains and unstained surfaces. Among these 42 rare microbial members, *Mycobacterium*, *Sphingobium*, *Phyllobacterium*, and *Vicinamibacter* (known as degraders of polycyclic aromatic hydrocarbons or chloroaromatic compounds [(57–59]) have been identified, which might be relevant for the biodegradation of black melanin pigments in stains.

## DISCUSSION

**Fig 6:**
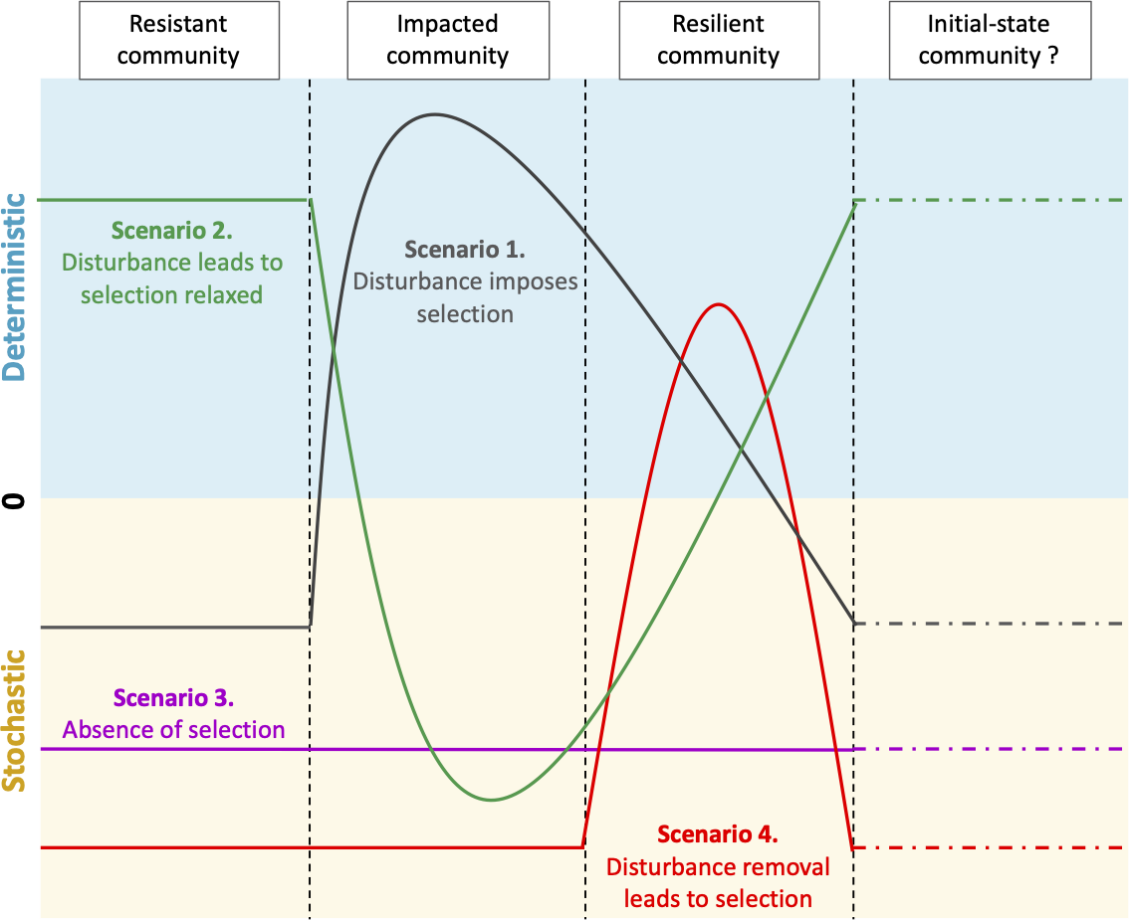
Four-scenario conceptual model integrating the three domains of life along the stochastic-deterministic continuum. The neutral hypothesis model (stochastic processes) and niche-based theory (deterministic processes) are represented in yellow and blue, respectively. Scenarios 1, 2, 3, and 4 are represented in black, green, purple, and red, respectively.

In the present work, metabarcoding data showed that the different visual states for rock surfaces corresponded to distinct microbial communities. Therefore, these rock surface conditions were appropriate to assess microbial particularities in relation to perturbation (leading to stain formation) and resilience (leading to stain attenuation). Thus, the response of microbial communities to disturbance was assessed in relation to predictive scenarios by explicitly considering the ecological differences between conventionally defined taxa categories (55, 56, 60), i.e., dominant taxa, moderate taxa, conditionally rare taxa, and always-rare taxa, and the assembly processes identified (Fig. 6). The thresholds for the operational definition of these categories are arbitrary, but the use of the same thresholds in different research is important to compare microbial assembly processes in different environments and investigations (27). Interestingly, several scenarios proved relevant to describe microbial responses in the current work.

### Scenario 1: disturbance imposes selection

In the resistant state (unstained surface), stochastic assembly processes (neutral theory) dominated. In the impacted state (black stains), deterministic assembly processes became predominant but lessened in the resilient state (attenuated stains). This scenario was demonstrated for Bacteria (considering all taxa together, or only the dominant taxa or the conditionallyrare taxa) and microeukaryotes (for dominant taxa and conditionally rare taxa). These results are conceptually consistent with previous findings on macroorganisms (in the cases of drought in ponds [7] and forest evolution after fire disturbance [61]) and microorganisms ([e.g., bacterial succession within pH soil gradients or across microbial desiccation processes [16, 62]).

### Scenario 2: disturbance leads to selection relaxed

Here, resistant communities (unstained surface) have undergone strong variable selection. Indeed, the disturbance weakens selection, which will lead to stochastic community assembly. As resilience develops (from black stains to attenuated stains), deterministic processes will increase in importance, as found for Archaea (all taxa together). The relevance of this scenario is supported by the analysis of forest harvesting (8, 63), which leads to the depletion of soil organic matter and soil compaction. Hartmann et al. (8) found that soil microbial communities were significantly different between such disturbed forests and non-harvested forests, even 15 years after harvest. Such a scenario was also observed for bacterial communities in agricultural soils affected by tillage (63).

### Scenario 3: absence of selection

This scenario reflects the neutral theory and highlights that some microbial systems are constantly dominated by stochastic processes (15). This might be linked to abundant resource supply and high levels of organismal dispersal (2). In the present work, this was the case for microeukaryotic dominant taxa. This concept has been previously reported in fluidic systems with constant resource supply (63), where only a few microbial taxa were prevented from growing.

### Scenario 4: disturbance removal leads to selection

Unexpectedly, the first three scenarios proved insufficient to depict the diversity of microbial responses documented. Therefore, it resulted in the proposition of a novel scenario applicable to the always-rare taxa. In this fourth scenario, the resistant and impacted states (unstained surface and black stain, respectively) displayed strong stochastic signatures, probably because perturbation was either minor for all taxa including always-rare taxa (in the resistant state) or strong for dominant taxa, thereby alleviating competition toward always-rare taxa (in the impacted state). In the resilient state, selection processes are important for always-rare taxa, perhaps because dominant taxa become better adapted and impose selection pressures. Alternatively, certain always-rare taxa may be positively selected by trophic opportunities, such as the fungus *Ochroconis lascauxensis* able to degrade benzalkonium chloride previously used in chemical treatments in Lascaux Cave (38, 64–66). Probably, this also includes *Mycobacterium*, *Sphingobium*, *Phyllobacterium,* and *Vicinamibacter*, which can degrade polycyclic aromatic hydrocarbons or chloroaromatic compounds that have a chemical structure similar to those of benzalkonium chloride and the benzene core of melanins. Therefore, these taxa may be involved in the biodegradation of, respectively, the chemical biocides used in Lascaux and the black pigments present in the stains (57–59). A previous rhizosphere study showed that rare bacterial taxa had undergone strong selection processes as well (20). Importantly, we found that scenario 4 was relevant for always-rare taxa from the three domains of life, suggesting common ecological behaviors as well as cooperative relations promoted by disturbance. It is probably of major significance for ecosystem functioning.

To conclude, we propose a novel scenario to predict the dynamics of always-rare taxa, which was validated in the case of Lascaux’s Chamber of Felines. We showed the necessity to consider the rare biosphere (and its functions) to fully assess microbial responses to environmental change and their involvement in ecosystem functioning. The conceptual model reported in this work (scenario 4) needs now to be tested in diverse environments to better predict and model ecosystem functioning.

## MATERIALS AND METHODS

### Sampling

Lascaux Cave is located in the south-west of France (N 45°03′13.087″ and E 1°10′12.362″). It received up to 1,800 visitors daily, until the cave closure in 1963 (49). Samples were collected in February 2017 and June 2022 from the walls of the Chamber of Felines (never visited by tourists), in areas devoid of Paleolithic paintings and engravings, with special authorization (from the Ministry of Culture, DRAC Nouvelle-Aquitaine). Six black stains, six control zones (i.e.*,* unstained surface), and six attenuated stains were sampled using swabs gently rubbed against the rock wall, so as to target wall surface microorganisms. For each replicate, samples for the three conditions were taken in the same location, at about 10–20 cm from one another, and the six locations were 1–2 m from one another. Strain attenuation did not proceed further between 2017 and 2022. Samples were conserved at −80°C until DNA extraction.

### Illumina sequencing and bioinformatics analysis

Total genomic DNA extraction was performed using the FastDNA SPIN Kit for Soil (MP Biomedicals, Illkirch, France), following the manufacturer’s instructions. DNA concentration was quantified using the Qubit dsDNA HS Assay Kit (Invitrogen, Carlsbad, CA, USA) following the manufacturer’s preconization.

Three gene markers were analyzed in each individual sample. The V3–V4 region of the 16S rRNA genes was amplified in triplicate for Bacteria and Archaea using the universal primers 341F (CCTACGGGNGGCWGCAG) and 805R (GACTACHVGGGTATCTAATCC) (67), and 515F (CAGCCGCCGCGGTAA) and 915R (GTGCTCCCCCGCCAATTCCT) (68), respectively. For Eukaryota, the V4 region of the 18S rRNA genes was amplified in triplicate using the universal primers 0067a_deg (AAGCCATGCATGYCTAAGTATMA) and NSR399 (TCTCAGGCTCCYTCTCCGG) (69). The PCR mix consisted of 5 µL of 5× Hot BioAmp Blend Master Mix RTL (Biofidal, Vaux-en-Velin, France), 0.1 µM of the two primers, 0.1× GC-rich-enhancer (Biofidal), 0.2 ng·µL^−1^ of bovine serum albumin (Promega, Madison, WI, USA), and 0.2–1.0 ng DNA. All amplifications were performed in a Bio-Rad T1000 thermal cycler (Bio-Rad, Hercules, CA, USA). The PCR program for Bacteria was 3 min at 95°C, 28 cycles of 45 s at 95°C, 45 s at 50°C, and 90 s at 72°C, followed by 7 min at 72°C. For Archaea, it consisted of 10 min at 94°C, 30 cycles of 1 min at 94°C, 1 min at 58°C, and 90 s at 72°C, followed by 10 min at 72°C. For the 18S rRNA gene, it consisted of 10 min at 95°C, 30 cycles of 1 min at 94°C, 1 min at 55°C, and 90 s at 72°C, followed by 10 min at 72°C. For the ITS2 region, it consisted of 10 min at 95°C, 28 cycles of 20 s at 94°C, 30 s at 47°C, and 20 s at 72°C, followed by 7 min at 72°C. All primers were tagged with the Illumina adapter sequences (TCG TCG GCA GCG TCA GAT GTG TAT AAG AGA CAG and GTC TCG TGG GCT CGG AGA TGT GTA TAA GAG ACA G), allowing the construction of amplicon libraries by a two-step PCR. DNA extraction was also carried out from tubes without any biological matrix, and this negative control was used to evaluate and subtract ambient and kit products’ contamination. Amplification signals were checked by electrophoresis on 1.5% agarose gel. High-throughput sequencing was achieved after pooling PCR triplicates, using Illumina MiSeq with 2 × 300 bp, paired-end chemistry v3 (aiming at 40,000 sequences for Bacteria and microeukaryotes, and 70,000 sequences for Archaea).

For each of the three datasets (i.e.*,* bacterial 16S rRNA genes, archaeal 16S rRNA genes, and eukaryotic 18S rRNA genes), paired-end reads were merged with a maximum of 10% mismatches in the overlap region using Fast Length Adjustment of Short reads (70). Denoising procedures were carried out by discarding reads without the expected length (200–500 bp) or containing any ambiguous bases (N). After the dereplication of sequences, the clusterization tool was run with Swarm (71), which uses a local clustering threshold rather than a global threshold and an aggregation distance of 3 for the identification of OTUs. Chimeric OTUs were removed using VSEARCH (72). Moreover, low-abundance sequences were filtered to keep OTUs representing at least 0.005% of all sequences (73). Taxonomic affiliation was performed with both RDP Classifier and BLASTn (74) against the 138.1 SILVA database (75) for bacterial 16S rRNA genes, archaeal 16S rRNA genes, and microeukaryotic 18S rRNA genes and against the 8.2 UNITE database for fungal ITS markers (76). This procedure was automated in the FROGS pipeline (77). Contaminant OTUs identified from the negative control samples were removed. To compare samples, a normalization procedure, using phyloseq package, was applied by randomly resampling down to 12,105, 6,209, 31,981, and 25,821 sequences in the bacterial, archaeal, microeukaryotic, and fungal data sets, respectively (78).

Rarefaction curves were calculated to assess sequencing effort, using Paleontological Statistics software v4.02 (79). OTU richness and diversity were estimated using Chao 1 index (80), Shannon’s H’ (81), and Simpson (1-D) index (82). Communities were primarily compared with NMDS, using vegan and stats packages in R (83–85). The procedure computes a stress value, which measures the difference between the ranks on the ordination configuration and the ranks in the original similarity matrix for each replicate (86). Analysis of similarity was conducted using the vegan package in R (83) to test the differences (*P* < 0.05) in overall community composition between the three rock surface conditions, i.e.*,* resistant, impacted, and resilient, and to further confirm the results observed in the NMDS plot. Multiple comparisons of treatments by Tukey’s test were performed using agricolae package. A Bonferroni correction was applied to *P* values to lower alpha risk. All analyses were based on abundance dissimilarity matrices with the Bray-Curtis index (87) using R.

### Definition of abundant and rare taxa

In this study, OTUs were classified, after combining data from both sampling dates, into four categories according to their relative abundance, as proposed by Liu et al*.* (55), used in subsequent works (24–27, 56, 60) and described in Fig. S5: (i) always-rare taxa, with an abundance < 0.01% in all samples; (ii) conditionally rare taxa, with an abundance < 1% in all samples and <0.01% in some samples (maximum 33% of samples); (iii) moderate taxa, with an abundance < 1% and >0.01% in all samples; and (iv) dominant taxa, with an abundance ≥ 1% of all sequences in all samples or that fluctuates strongly, i.e.*,* <0.01% in certain samples and ≥1% in others. Metagenomic data for other Lascaux rooms suggest that artificial over-representation of certain microbial groups with the molecular approach followed could concern taxa already prominent and was very unlikely to have concerned rare taxa.

### Assessment of community dispersal rate: neutral model

Sloan’s NCM was used to estimate the importance of passive dispersal on community assembly by assessing the relationship between the frequency at which taxa occur in a set of local communities and their abundance across the wider meta-community (4). In this model, the migration rate (*m*) is a parameter used to evaluate the probability of a random loss of an individual in a local community, to be replaced by an immigrant from the meta-community (3). It is calculated as follows:


$$Freq_{i}=1-I\left(1÷N|N\times m\times p_{i},N\times m\times \left(1-p_{i}\right)\right),$$

where Freq*_i_* is the occurrence frequency of taxa *i* across communities; *N* is the number of individuals per community; *m* is the estimated dispersal rate; *p_i_* is the average relative abundance of taxa *i* across communities; and *I*() is the probability density function of β-distribution (21). This analysis was performed using nonlinear least-square fitting, and calculation of 95% confidence interval for the model predictions was conducted using the Wilson score interval (“minpack.lm” and “Hmisc” package in R [88, 89]). The overall fit of the model to observed data was done by comparing the sum of squares of residuals (SS_err_) with the total sum of squares (SS_total_), with model fit = 1 − SS_err_/SS_total_ (generalized R-squared). The Akaike information criterion and Bayesian information criterion (BIC) were calculated based on 1,000 bootstrap replicates for the neutral, binomial, and Poisson distribution models to determine the best model fit and whether the model was based on only the random sampling of the source meta-community.

### Phylogenetic analysis and estimation of assembly community processes

In order to determine the ecological processes controlling the assembly of all taxa and the different abundance categories of taxa described above (dominant taxa, moderate taxa, conditionally rare taxa, and always-rare taxa), phylogenetic trees were built independently for each of the five categories and for each taxonomic marker (in total, 15 different phylogenetic trees). Briefly, for each taxonomic marker, sequences were aligned using MAFFT (90), then gaps and poorly conserved regions were eliminated using Gblocks (91, 92). Finally, phylogeny was reconstructed using IQ-TREE-2 (93) (default parameters), with the best model being automatically chosen by the program according to the BIC. Node support was estimated using the bootstrap method (1,000 replicates).

To quantify the relative importance of stochastic and deterministic processes that drive microbial community assembly, βNTI and RC_bray_ were calculated using ade4, picante, and iCamp packages and scripts from Stegen et al. in R software v4.0.2 (13, 94–96), based on the phylogenetic distance and OTU abundance (*n* > 6 OTUs) (13). βNTI is the number of standard deviations of the β-mean nearest taxon distance from the mean of the null distribution (13). The βNTI is the difference between the observed β-mean nearest taxon index and the mean of the null distribution of βMNTD normalized by its standard deviation. The βNTI values between –2 and 2 indicate the dominance of the stochastic processes, whereas βNTI values smaller than –2 or larger than 2 indicate that deterministic processes (i.e., homogeneous selection and heterogeneous selection) play a more important role in community assembly than stochastic processes (2). Indeed, βNTI values > +2 indicate significantly more phylogenetic turnover than expected (heterogeneous selection), and βNTI values < −2 less indicate phylogenetic turnover than expected (homogeneous selection) (53). As the observed βMNTD value did not significantly deviate from the null βMNTD distribution (i.e.*,* |βNTI| < 2), it meant stochastic turnover in phylogenetic composition. Since this stochasticity could entail dispersal limitation, homogenizing dispersal, or the lack of a single dominant process (termed the undominated fraction) (13, 97), this was clarified by calculating the RC_bray_ on pairwise comparisons with |βNTI| < 2, as it enables to further partition pairwise comparisons that were assigned to stochastic processes (2). Thus, for |βNTI| < 2, RC_bray_ < –0.95 and RC_bray_ > 0.95 indicate the relative dominant influence of homogenizing dispersal and dispersal limitation, respectively, and |RC_bray_| < 0.95 indicates a crucial role of “undominated” assembly, including weak selection, weak dispersal, diversification, and/or drift (2, 13, 14, 54). The major factors that influence the assembly of dominant taxa, conditionally rare taxa, and always-rare taxa were explored separately.

## Data Availability

All data that support the findings of this study have been deposited in EBI under reference PRJEB46483, PRJEB46545, and PRJEB46546 for bacterial 16S rRNA genes, archaeal 16S rRNA genes, and microeukaryotic 18S rRNA genes, respectively. The authors declare that the R (R 4.0.2) codes used to generate the results reported in this study are available in this paper. The R code supporting the finding presented here is available from the GitHub Repository https://github.com/LascauxZelia/Bontemps_et_al_2022.

## SUPPLEMENTAL MATERIAL

Supplemental material msystems.01233-23-s0001.pdf Supplemental figures and tables.

ASM does not own the copyrights to Supplemental Material that may be linked to, or accessed through, an article. The authors have granted ASM a non-exclusive, world-wide license to publish the Supplemental Material files. Please contact the corresponding author directly for reuse.
