# Supplementary material for: Stochastic and deterministic assembly processes of microbial communities in relation to natural attenuation of black stains in Lascaux Cave
Source: mSystems. 2024 Jan 30;9(2):e01233-23. doi: 10.1128/msystems.01233-23 (PMC10878041; doi:10.1128/msystems.01233-23)
Supplement: Supplemental material — Supplemental figures and tables. [file msystems.01233-23-s0001.pdf]

Supplementary information

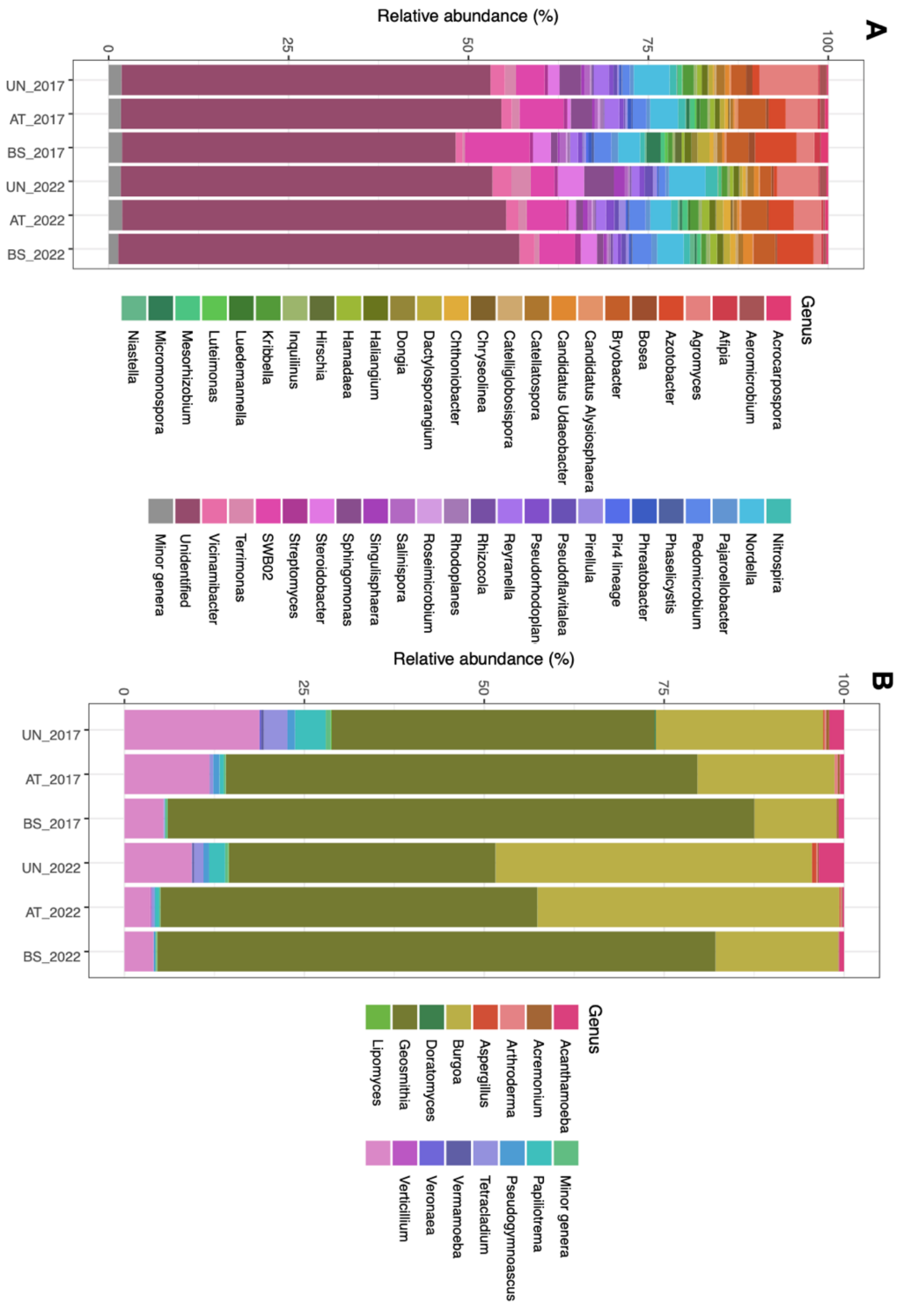

Supplementary Figure 1. Comparison at genus level of microbial communities on unstained limestone (resistant microbial community), black stain (impacted community) and attenuated stain (resilient status) in Lascaux's Chamber of Felines. The analysis was done with bacteria **(A)** and microeukaryotes **(B)**. Genera representing less than 0.1% are combined in the Minor genera category.

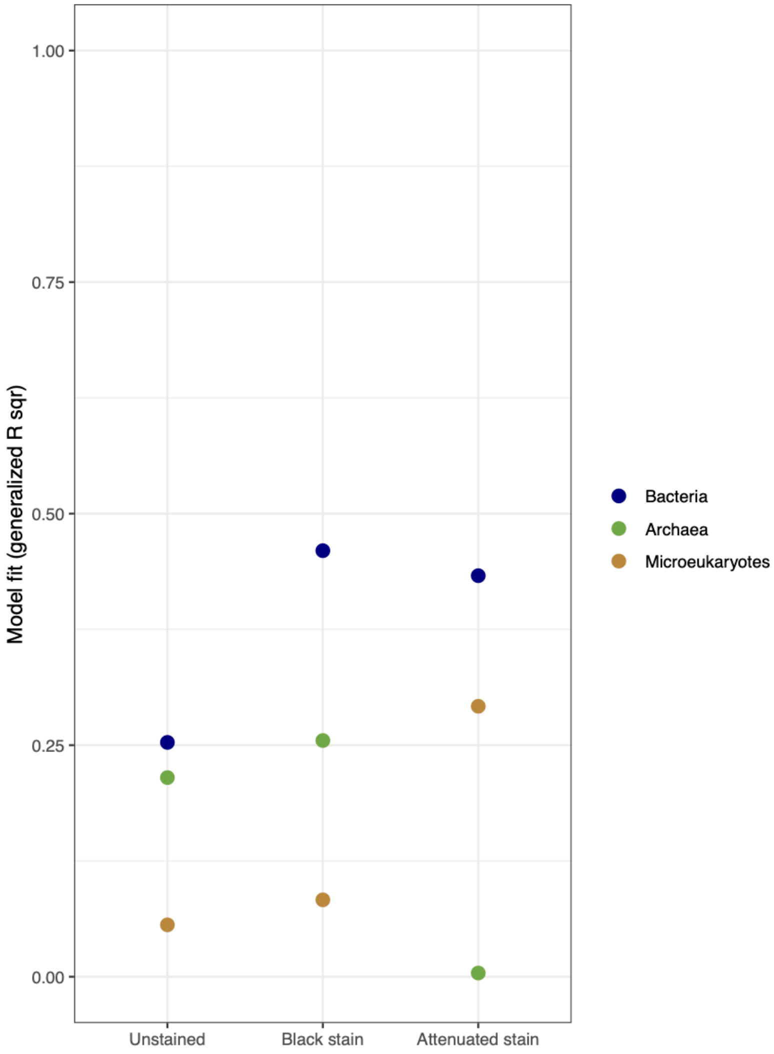

Supplementary Figure 2. The goodness-of-fit of the neutral model during microbial dynamics for the three domains of life.

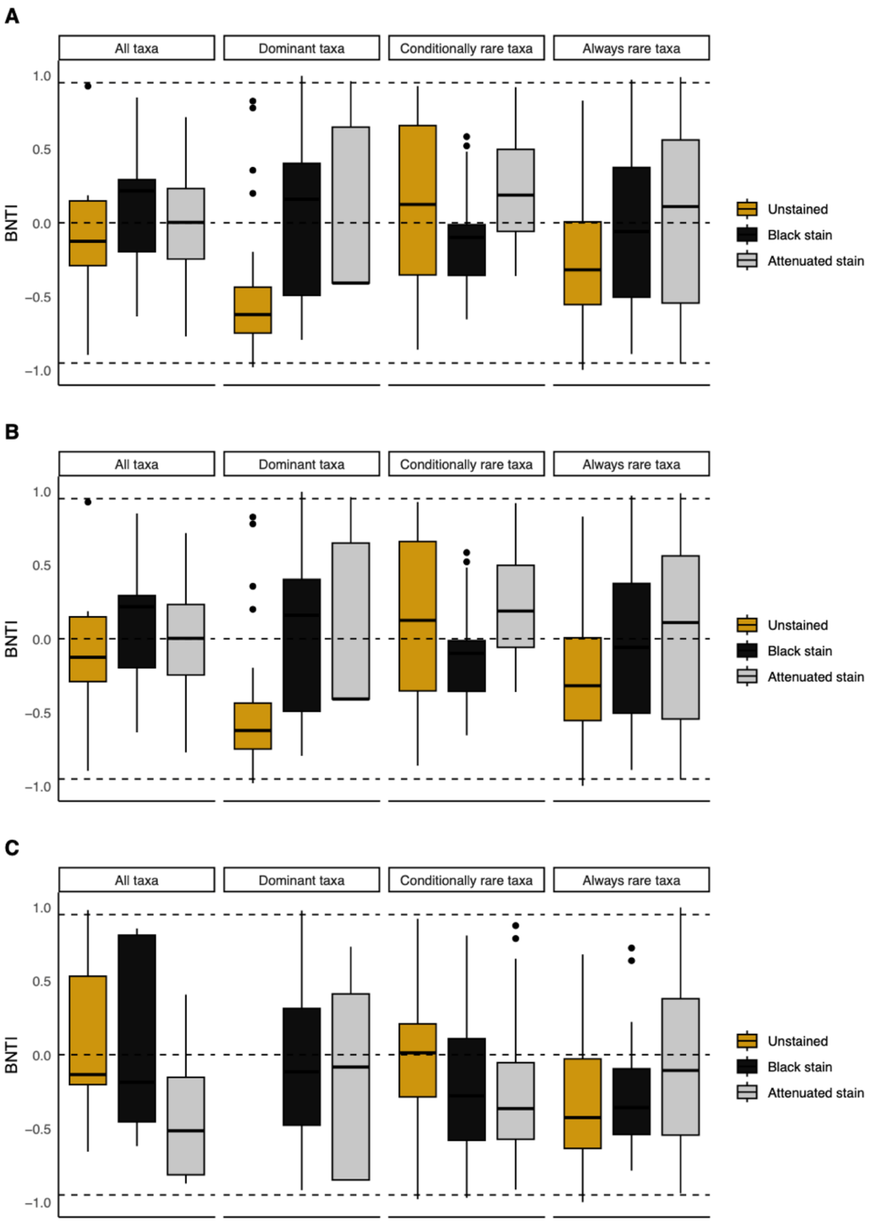

Supplementary Figure 3. βNTI patterns for bacterial, archaeal and microeukaryotic communities across rock surface conditions. Horizontal dashed lines indicate null values as well as upper and lower significant thresholds of -2 and +2. In **B**, no data is shown for dominant taxa and conditionally-rare taxa because the number of OTUs in these categories was low (< 6).

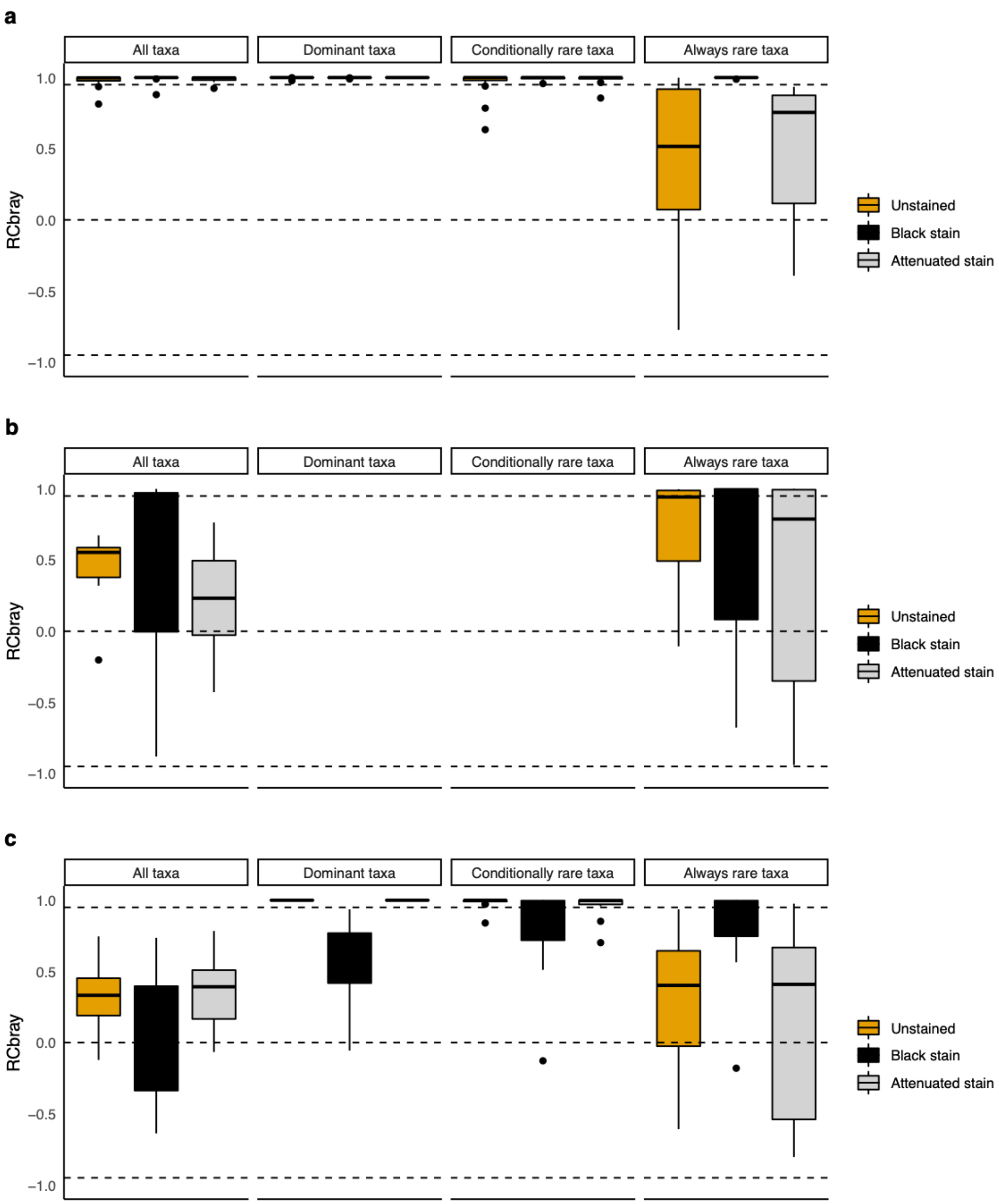

Supplementary Figure 4. Bray-Curtis-based Raup-Crick (RC_{bray}) values for bacterial, archaeal and microeukaryotic communities across rock surface conditions. Horizontal dashed lines indicate null values as well as upper and lower significant thresholds of -0.95 and +0.95. In **B**, no data is shown for dominant taxa and conditionally-rare taxa because the number of OTUs in these categories was low (< 6).

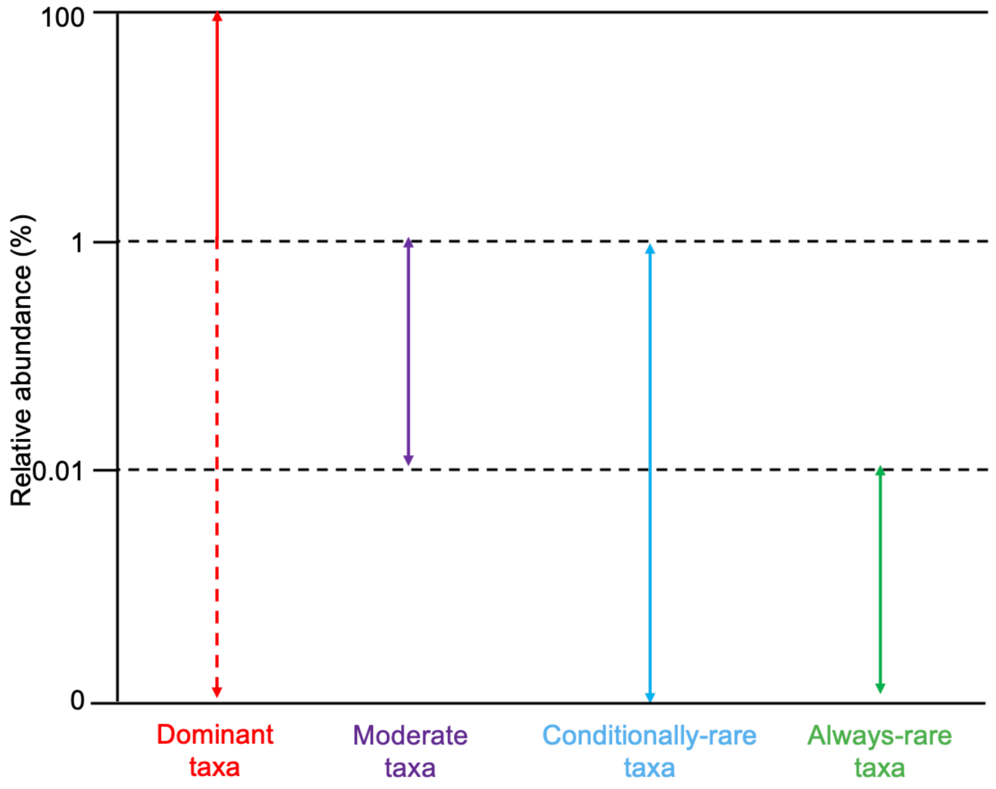

Supplementary Figure 5. Thresholds used to define abundant and rare taxa categories in this study. Dominant taxa were (i) OTUs with a relative abundance between 1% and 100% in all samples or (ii) OTUs with a relative abundance varying from rare (< 0.01%) to abundant (≥ 1%). Moderate taxa were the OTUs with relative abundance between 0.01% and 1% in all samples. Conditionally-rare taxa were the OTUs with a relative abundance < 0.01% in no more than 33% of all samples but never ≥ 1% in all samples. Always-rare taxa were the OTUs with a relative abundance < 0.01% in all samples.

Supplementary Table S1. Effect of rock surface condition (unstained surface, black stain, attenuated stain) and sampling time (2017, 2022) on microbial community composition.

Gene	Effect	Df	F	*P*	R^2
Bacterial 16S rRNA	Status	2	2.874	**0.001**	0.25
	Time	1	3.001	0.640	0.02
	Residuals	15			
Archaeal 16S rRNA	Status	2	1.324	**0.035**	0.14
	Time	1	1.321	0.113	0.01
	Residuals	15			
Microeukaryotic 18S rRNA	Status	2	2.649	**0.001**	0.25
	Time	1	1.435	0.095	0.04
	Residuals	15			

Supplementary Table S2. Diversity indices for bacterial, archaeal and microeukaryotic communities for each condition studied.

		Black stain 2017		Black stain 2022		Attenuated stain 2017		Attenuated stain 2022		Unstained surface 2017		Unstained surface 2022	
		Mean	SD	Mean	SD	Mean	SD	Mean	SD	Mean	SD	Mean	SD
16S Bacteria	Chao1	411.3	61.9	479.8	47.0	484.3	59.4	544.0	27.2	449.9	37.7	537.6	31.4
	Shannon	4.29	0.29	4.20	0.19	4.31	0.47	4.48	0.23	4.15	0.36	4.36	0.26
	Simpson	32.06	10.15	26.54	5.46	31.21	16.35	35.48	14.43	26.78	16.66	29.43	13.62
16S Archaea	Chao1	37.6	10.5	32.9	10.1	41.2	15.9	39.2	7.9	37.2	7.0	48.3	10.8
	Shannon	1.29	0.68	0.61	0.34	1.37	0.29	1.07	0.40	0.84	0.41	1.42	0.20
	Simpson	2.87	1.65	1.44	0.40	2.77	0.78	2.23	0.89	1.74	0.67	2.87	0.43
18S Microeukaryotes	Chao1	67.4	23.3	68.5	9.2	93.2	18.4	85.9	13.7	106.9	16.9	113.1	14.1
	Shannon	0.63	0.46	0.70	0.23	1.18	0.51	1.01	0.18	1.82	0.05	1.51	0.18
	Simpson	1.50	0.61	1.57	0.36	2.22	0.98	2.11	0.30	3.26	0.70	2.70	0.48

Supplementary Table S3. Comparison of the maximum likelihood fit of the neutral, binomial and Poisson models for each gene and each rock surface condition. *m*: estimated migration rate; AIC: Akaike Information Criterion; BIC: Bayesian Information Criterion; BS: Black stain; AT: Attenuated stain; UN: Unstained; All: All rock surface conditions.

Gene	Zone	*M*	R^2 neutral	R^2 binomial	R^2 Poisson	AIC neutral	AIC binomial	AIC Poisson	BIC neutral	BIC binomial	BIC Poisson
Bacterial 16S rRNA	BS	0.207	0.460	-0.405	-0.405	-106.9	-84.12	-84.00	-98.01	-75.22	-75.00
	AT	0.493	0.433	0.005	0.005	-339.9	-279.9	-279.9	-330.3	-271.0	-271.0
	UN	0.431	0.253	-0.131	-0.131	-147.9	-42.71	-42.71	-139.0	-33.81	-33.81
	All	0.294	0.175	-0.309	-0.309	-102.5	-21.63	-21.60	-201.0	-74.21	-74.22
Archaeal 16S rRNA	BS	0.051	0.255	-1.000	-1.000	47.71	625.7	625.7	52.08	630.1	630.1
	AT	0.108	0.004	-0.723	-0.723	32.02	42.55	42.55	36.24	46.77	46.75
	UN	0.340	0.215	-0.147	-0.147	-3.501	22.61	22.61	-18.45	0.654	0.650
	All	0.154	0.102	-0.076	-0.076	10.08	31.80	31.80	1.754	67.98	67.98
Microeukaryotic 18S rRNA	BS	0.022	0.083	-2.779	-2.779	47.71	625.7	625.7	52.03	630.1	630.0
	AT	0.068	0.292	-1.558	-1.558	42.52	320.0	322.0	46.77	66.24	66.25
	UN	0.206	0,056	-0.579	-0.579	-22.61	-3.501	-3.544	-18.46	0.654	0.691
	All	0.061	0.359	-0.465	-0.465	-19.15	14.90	14.87	-5.486	18.46	18.56
